# Aging is associated with highly defined epigenetic changes in the human epidermis

**DOI:** 10.1186/1756-8935-6-36

**Published:** 2013-10-31

**Authors:** Günter Raddatz, Sabine Hagemann, Dvir Aran, Jörn Söhle, Pranav P Kulkarni, Lars Kaderali, Asaf Hellman, Marc Winnefeld, Frank Lyko

**Affiliations:** 1 Division of Epigenetics, DKFZ-ZMBH Alliance, German Cancer Research Center, Im Neuenheimer Feld 580, Heidelberg 69120, Germany; 2 Research & Development, Beiersdorf AG, Unnastrasse 48, Hamburg 20253, Germany; 3 Institute for Medical Research Israel-Canada, Hebrew University-Hadassah Medical School, Jerusalem 91120, Israel; 4 Institute for Medical Informatics and Biometry, Dresden University of Technology, Fetscherstrasse 74, Dresden 01307, Germany

**Keywords:** Aging, DNA methylation, Epidermis, Methylome sequencing, Transcriptome sequencing

## Abstract

**Background:**

Altered DNA methylation patterns represent an attractive mechanism for understanding the phenotypic changes associated with human aging. Several studies have described global and complex age-related methylation changes, but their structural and functional significance has remained largely unclear.

**Results:**

We have used transcriptome sequencing to characterize age-related gene expression changes in the human epidermis. The results revealed a significant set of 75 differentially expressed genes with a strong functional relationship to skin homeostasis. We then used whole-genome bisulfite sequencing to identify age-related methylation changes at single-base resolution. Data analysis revealed no global aberrations, but rather highly localized methylation changes, particularly in promoter and enhancer regions that were associated with altered transcriptional activity.

**Conclusions:**

Our results suggest that the core developmental program of human skin is stably maintained through the aging process and that aging is associated with a limited destabilization of the epigenome at gene regulatory elements.

## Background

Epigenetic mechanisms regulate the interpretation of genetic information and are thus intricately linked to cellular differentiation and tissue specification. Epigenetic mechanisms include DNA methylation and covalent histone modifications [1,2]. These mechanisms work in a coordinated manner during development and differentiation to execute specific gene expression programs [3,4].

DNA methylation is a conserved epigenetic mechanism with a well-known role in cell fate specification [5,6]. In mice, this function appears to be essential for organismal development, as genetic deficiencies for DNA methyltransferases cause severe developmental phenotypes and embryonic lethality [7,8]. More recently, significant attention has also been focused on adaptive functions of DNA methylation, which could provide the foundation for the integration of environmental signals [9]. Initially observed as epigenetic variations between monozygotic twins [10], age-related methylation differences have now been described in several independent studies and tissues [11]. The results suggested that aging induces global and complex changes in the human methylation landscape [12-15]. However, it is presently unclear how these epigenetic changes affect the gene expression patterns of aging human tissues.

We have previously used array-based methylation profiling of skin samples obtained from young and old volunteers and identified a defined set of genes that were hypermethylated in aged skin [16]. In the course of this study, two important features were identified that established the epidermis as an important tissue for further analysis: i) epidermis samples can be obtained by non-invasive procedures and are characterized by a very high degree of cell type homogeneity (>90% keratinocytes), which greatly facilitates the identification of defined methylation patterns; and ii) the epidermis represents a tissue with high functional relevance for human health and disease and shows a well-known aging phenotype [17]. Furthermore, it has also been shown that the DNMT1 DNA methyltransferase is functionally required for proper tissue homeostasis in the human epidermis [18].

Aging is a multifactorial process that results in a progressive loss of regenerative capacity and tissue functionality. It has previously been suggested that these pathological changes are underpinned by a genome-wide loss of methylation marks [15]. We have now used transcriptome sequencing to identify genes that are differentially expressed in the young (18–24 years) and old (70–75 years) human epidermis. This revealed a highly specific set of 75 genes with strong functional relevance for human skin homeostasis. We also used whole-genome bisulfite sequencing to generate methylation maps from the same tissue samples. We find that the aging epidermis methylome is characterized by considerable stability and does not show any global methylation loss. However, local age-related methylation changes could be observed, and were enriched at weak or poised promoters and at enhancer regions, thus suggesting a functional relevance of epigenetic mechanisms for skin aging.

## Results

### The transcriptome of the aging human epidermis

Primary human epidermis samples were obtained as suction blisters from the inner forearm of five female donors from two distinct age groups (18–24 years and 70–75 years, see Additional file 1: Table S1 for details), respectively. These two age groups provide a good representation of the skin aging phenotype in healthy adults [17]. After RNA purification, samples were pooled in equimolar ratios to allow library preparation for transcriptome sequencing. Pooling of samples was required to obtain sufficient amounts of mRNA for library preparation. Paired-end sequencing on an Illumina HiSeq 2000 platform with read-lengths of 105 bases generated 90 Gb of DNA sequence. After trimming to a maximal read length of 80 bases and a minimum base quality of a 30 Phred score, sequence reads were mapped to the GRCh37/hg19 reference sequence using TopHat [19]. The resulting average strand-specific genome coverages were 500x for each sample (Table 1).

**Table 1:** Sequencing data

**Sample**	**Age range**	**Sequencing**	**Number of read pairs**	**Mapping efficiency**	**Coverage**	**Conversion rate**
Young (n = 5)	18–24	Transcriptome	225,332,853	89%	500x	n. d.
		Methylome	344,185,333	69%	11.3x	99.84%
Old (n = 5)	70–75	Transcriptome	221,387,474	88%	500x	n. d.
		Methylome	334,873,828	74%	11.9x	99.88%

Coverage indicates the average strand-specific genome or transcriptome coverage. Bisulfite conversion rates were determined by analyzing the conversion rates of co-purified mitochondrial genomes that were considered as unmethylated. Mitochondrial genome coverages were 1319x (young) and 1643x (old), respectively. n. d.: not determined.

Overall, the epidermis transcriptome was characterized by high expression levels of epidermis-specific genes, such as various epidermis keratins and the epidermis structural genes Loricin and Filaggrin (Table 2). Genes that are predominantly expressed in cells of mesenchymal origin, such as Vimentin and Desmin, could not be detected or were only expressed at low levels (Table 2), which is in agreement with the substantial cell-type homogeneity of the samples used for this study. The expression patterns of these genes appeared highly similar in the young and old epidermis (Table 2), thus reflecting the stability of the core epigenetic program of differentiated human tissues. In agreement with this notion, gene expression levels for DNA methylation enzymes, such as DNMTs and TETs and their known cofactors DNMT3L and UHRF1, were low and also appeared very similar in the old and young samples (Table 2).

**Table 2:** Expression levels of epidermis genes and DNA methylation factors

**Gene group**	**Gene**	**Expression (young)**	**Expression (old)**	**q value**
Epidermis keratins	*KRT1*	14719.2	14954.2	1.0
	*KRT2*	7291.7	6154.7	1.0
	*KRT5*	5860.6	5247.7	1.0
	*KRT10*	26232.1	23374.7	1.0
	*KRT14*	5665.0	4430.4	1.0
	*KRT15*	382.1	400.1	1.0
Epidermis structural factors	*LOR*	613.4	427.3	1.0
	*FLG*	558.1	501.3	1.0
	*FLG2*	322.5	336.5	1.0
Fibroblast markers (dermis)	*VIM*	41.0	54.0	1.0
	*DES*	<0.1	<0.1	1.0
DNA methylation factors	*DNMT1*	9.5	7.3	1.0
	*DNMT3A*	1.5	1.2	1.0
	*DNMT3B*	1.4	1.5	1.0
	*DNMT3L*	<0.1	<0.1	1.0
	*UHRF1*	1.3	1.2	1.0
	*TET1*	0.1	0.1	1.0
	*TET2*	8.4	6.9	1.0
	*TET3*	18.8	14	1.0

Expression levels represent FPKM values [21]; levels >1 indicate expression; q values were calculated by DESeq [20].

**Figure 1:**
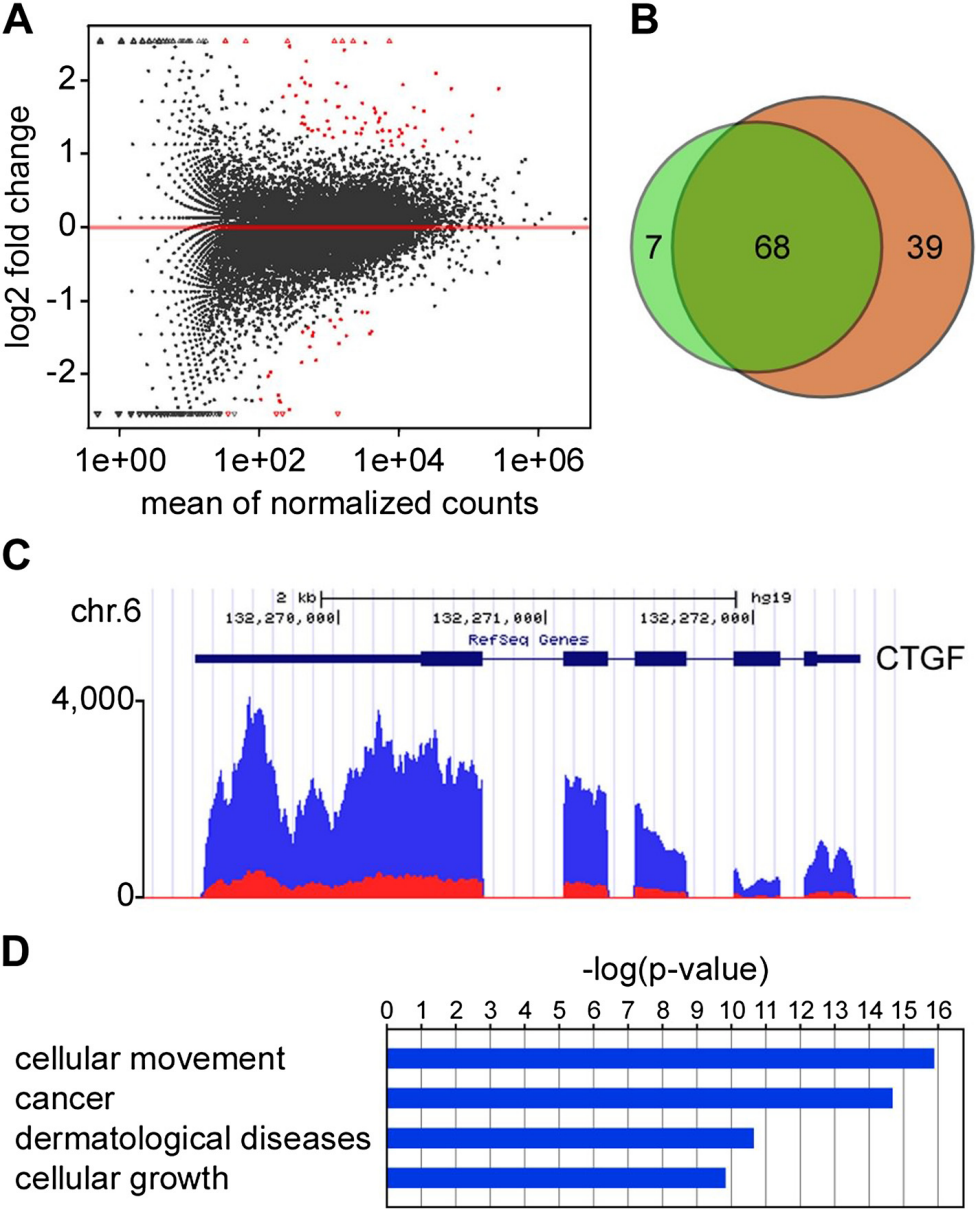
**The transcriptome of the aging human epidermis. (A)** MA plot showing fold change between young and old in relationship to the mean expression level. Red marks indicate genes with significant differential expression, as determined by DESeq analysis. **(B)** Venn diagram showing the overlap in differentially expressed gene sets, as determined by DESeq (green) and by Cuffdiff 2 (orange). **(C)** Connective Tissue Growth Factor (CTGF) is shown as a representative example for a differentially expressed gene. RNA-seq coverage is indicated for young (blue) and old (red) samples. **(D)** Ingenuity pathway analysis of differentially expressed genes. The plot shows the four most significantly enriched functional categories.

To systematically identify genes that were differentially expressed in the young and the old sample, we used DESeq [20]. This revealed 75 genes with a statistically significant difference (Q <0.05, Additional file 2: Table S2), suggesting that the young and old epidermis transcriptomes were similar overall. This similarity was also confirmed by the relatively small effect size of age-related differential gene expression (Figure 1A). We also used an independent algorithm for differential expression analysis, Cuffdiff 2 [21]. This identified an overall similar number of 107 differentially expressed genes. Notably, out of the 75 genes identified by DESeq, 68 were also identified by Cuffdiff 2 (Figure 1B). This substantial overlap suggests that aging of the human epidermis affects the expression of a specific set of genes.

Further analysis of differentially expressed genes identified several factors that have previously been implied in skin homeostasis. For example, connective tissue growth factor (CTGF), which has been shown to be an important factor in skin aging [22], was expressed at distinctly lower levels in the old epidermis (Figure 1C). We also used pathway analysis of differentially expressed genes to further characterize the functional effects of age-related differential gene expression. This revealed that genes involved in cell migration (*P* = 1.17 × 10^-16^), cancer (*P* = 9.55 × 10^-13^), dermatological diseases (*P* = 2.07 × 10^-11^) and cell proliferation (*P* = 1.4 × 10^-10^), constituted the categories with the most significant enrichment (Figure 1D). In addition, functional annotation of differentially expressed genes also showed a highly significant enrichment of genes involved in the development of the epidermis (*P* = 9.63 × 10^-4^), which also included downregulation of SPRR1A and B, KRT16 and KRT17 in old samples. In agreement with this finding, expression of genes involved in differentiation of keratinocytes was also altered in old samples, indicating an overall downregulation of differentiation (*P* = 1.43 × 10^-7^). Altogether, our results thus suggest that deregulation of a relatively small set of genes could contribute to the phenotypic changes associated with human skin aging.

**Figure 2:**
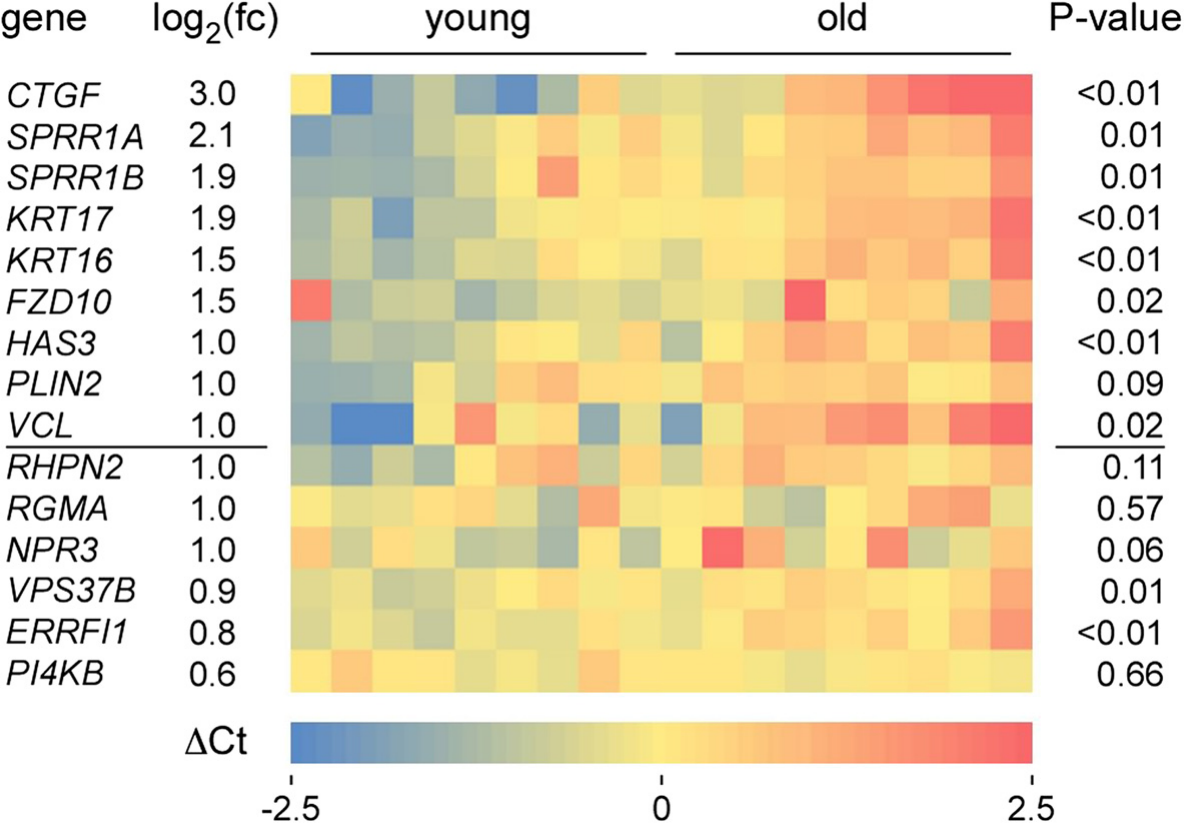
**Validation of differential age-related gene expression in individual tissue samples.** qRT-PCR was performed on RNA from epidermal suction blisters of 18 healthy female volunteers (9 young and 9 old volunteers). The heatmap shows processed ΔCt values. Gene expression differences of individual samples are indicated relative to the gene-specific, age-independent average expression level over all samples (blue: lower ΔCt, red: higher ΔCt). Numbers in the left colum indicate fold-change differences in gene expression between young and old, as determined by transcriptome sequencing. *P* values were determined by a Mann-Whitney U test and indicate the significance for differential gene expression. The line between *VCL* and *RHPN2* separates genes with a ≥2-fold change in gene expression from genes with a <2-fold change.

To further analyze the patterns of differential gene expression, we experimentally validated the differential expression levels of defined genes in 18 individual epidermis samples from the two age groups (9 young and 9 old). For these experiments, we selected 15 genes with a functional annotation related to skin homeostasis that had shown different degrees of age-related downregulation in our RNA-seq analysis. The results showed significant (*P* <0.05, Mann-Whitney U test) differential gene expression for 8 out of 9 genes that had shown a ≥2-fold expression change in our RNA-seq analysis (Figure 2). For genes with a fold change of <2, significant differential expression was only observed in 2 out of 6 genes (Figure 2). These findings provide important confirmation for our transcriptome sequencing results and suggest that age-related gene deregulation occurs with a substantial degree of population homogeneity.

### The methylome of the human epidermis

Having shown that aging is associated with the deregulation of a highly defined set of genes, we used whole-genome bisulfite sequencing to establish DNA methylation maps at single-base resolution. DNA was purified from the same epidermis samples that were used for transcriptome sequencing (Additional file 1: Table S1). Pooling of samples was necessary to achieve sufficient amounts of DNA for library preparation and has been previously used to reduce the effects of stochastic epigenetic variation [23]. Paired-end sequencing on an Illumina HiSeq 2000 platform with read-lengths of 105 bases generated 137 Gb of DNA sequence. After trimming to a maximal read length of 80 bases and a minimum base quality of a 30 Phred score, sequence reads were mapped to the GRCh37/hg19 reference sequence using a mapping tool based on BSMAP 2.0. The resulting average strand-specific genome coverage was 11.3x (young) and 11.9x (old). We also determined the bisulfite conversion rate by analyzing mitochondrial sequences that were co-purified during the sample preparation and that we considered as unmethylated. These sequences showed a bisulfite conversion rate of >99.8% (Table 1), suggesting highly effective bisulfite treatment.

**Figure 3:**
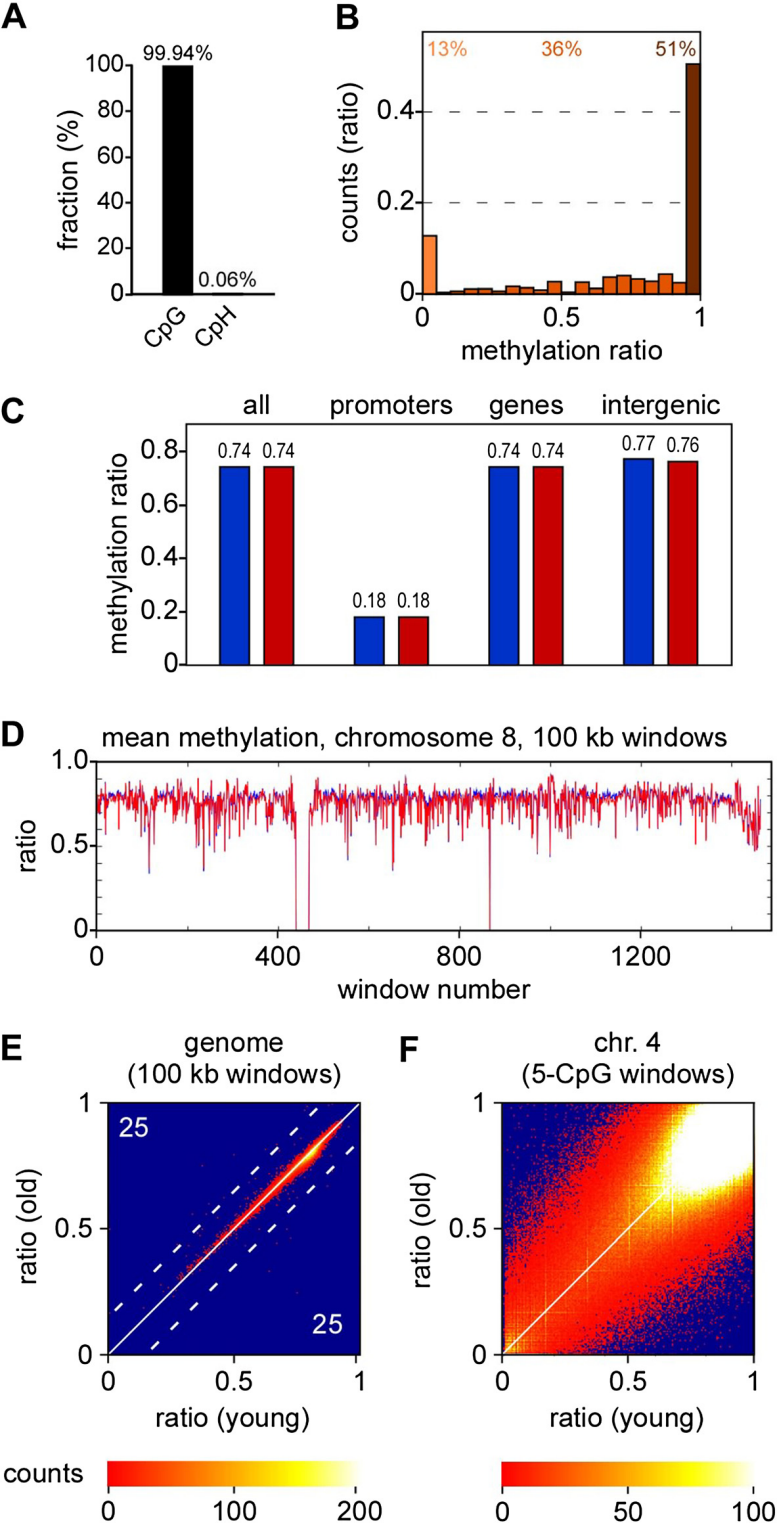
**The methylome of the aging human epidermis. (A)** Dinucleotide context of non-converted cytosine residues. **(B)** Methylation levels of individual CpG dinucleotides. Average methylation levels were determined for all covered CpG dinucleotides and then distributed into bins with increasing methylation ratios. Percentages indicate the fractions of unmethylated (light orange), partially methylated (orange) and completely methylated (dark orange) CpGs. **(C)** Average DNA methylation ratios of the genome (all), promoters, gene bodies and intergenic regions are shown for the young (blue) and old sample (red), respectively. **(D)** The methylation pattern of chromosome 8, shown in tracks of 100-kb windows. The blue line indicates the young sample, the red line indicates the old sample. **(E)** Density plot of average methylation ratios for 100-kb windows covering the entire genome. Numbers indicate the number of windows with a methylation difference >0.15 (dotted line). **(F)** Density plot of average DNA ratios for 5-CpG windows covering chromosome 4.

Initial data analysis revealed that the human epidermis shares many basal features with published epigenomes from differentiated cultured human cell lines [3,4,24,25]. For example, the vast majority (>99.9%) of non-converted cytosines were found in a CpG dinucleotide context (Figure 3A), which is consistent with the overall deamination efficiency and in agreement with the notion that non-CpG methylation is largely restricted to embryonic stem cells [24]. Furthermore, methylation ratios of individual CpG dinucleotides revealed a characteristic bimodal distribution (Figure 3B). A major fraction of CpG dinucleotides (about 50%) showed complete methylation, as indicated by a methylation ratio of >0.95 (Figure 3B). Roughly 10% of the CpGs were completely unmethylated (methylation ratio <0.05), while 40% of the CpG dinucleotides showed partial methylation ratios between 0.05 and 0.95 (Figure 3B). The average CpG methylation ratio was 0.74 (Figure 3C), which is again consistent with the CpG methylation ratios observed in other human datasets. Furthermore, average methylation ratios of promoter-associated CpGs were distinctly lower than the genome average, while gene bodies and intergenic regions showed higher methylation levels (Figure 3C), which is again similar to other published datasets.

Overall, the methylation patterns of the young and old samples appeared very similar. This was evident not only by the average methylation ratios of individual genome compartments (Figure 3C), but also in comparisons of the global methylation landscapes (Figure 3D). A sliding window approach identified only 50 differentially methylated windows of 100 kb (methylation difference >0.15), with an equal number of hypomethylated and hypermethylated windows (Figure 3E). Similarly, a more local analysis with sliding windows of 5 CpGs did not reveal any directional changes in global methylation patterns (Figure 3F). Together, these findings strongly suggest that the global age-related methylation loss observed in T-cells [15] is not conserved in the epidermis.

### Identification and characterization of differentially methylated regions

A visual inspection of the young and old methylation landscapes also indicated the presence of small clusters of differentially methylated CpG dinucleotides. To systematically identify differentially methylated regions (DMRs), Fisher’s exact test was used to determine the CpG dinucleotides with a statistically significant (*P* <0.05) methylation difference. These differentially methylated CpGs (DMCs) were subsequently collapsed to identify regions of local, coordinated methylation changes. DMRs were defined as clusters of ≥8 DMCs with a distance of ≤50 bp between neighboring DMCs and a net region-wide methylation change of ≥8 DMCs. Only DMRs with an average sequencing coverage of ≥8 and methylation difference of ≥10% were used for further analysis. This identified 2,409 DMRs, of which 1,437 were more strongly methylated in the old sample, and 972 were more strongly methylated in the young sample. DMRs were comparably small (<150 bp) and associated with various gene regions. Notably, 1,156 of these DMRs overlapped with the variably methylated regions that were recently identified through a comprehensive analysis of 42 human methylomes [26]. This represents a robust (2.5-fold) enrichment over the genome average and suggests that age-related methylation changes affect epigenetic regulatory elements.

**Figure 4:**
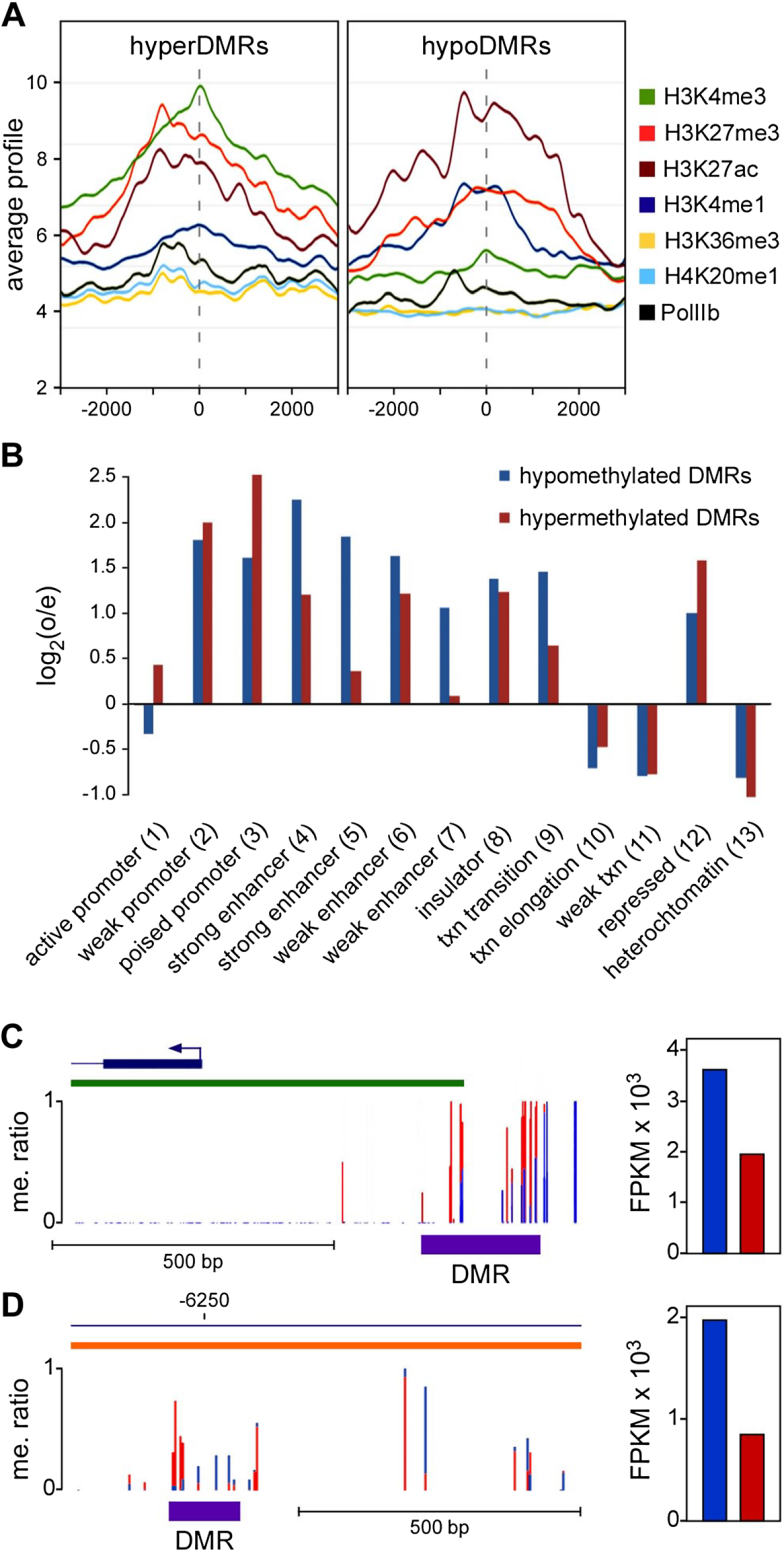
**Characterization of differentially methylated regions (DMRs). (A)** Hypermethylated and hypomethylated DMRs show distinct chromatin states. DMRs that become hypermethylated in old epidermis (hyperDMRs) are enriched for H3K4me3 and H3K27me3, while DMRs that become hypomethylated in old epidermis (hypoDMRs) are enriched for H3K27ac. Numbers below the x-axis indicate the distance from the center of the DMR (dashed vertical line) in bp. **(B)** Enrichment of DMRs within defined genome segments. Bars indicate the ratio of the observed DMR frequency and the average frequency across the genome. Blue bars represent DMRs that are hypomethylated in the old epidermis, red bars represent DMRs that are hypermethylated in the old epidermis. **(C)** The ERBB receptor feedback inhibitor 1 (ERRFI1) promoter region harbors a representative DMR. ERRFI1 is required for proper epidermal homeostasis [30] and is expressed at lower levels in old epidermis samples (right panel, blue and red bars indicates expression in the young and old epidermis samples, respectively). Blue lines indicate methylation ratios in the young epidermis, red lines indicate methylation ratios in the old epidermis. The green bar indicates the position of the ERRFI1 promoter CpG island. **(D)** An annotated active enhancer element from the low-density lipoprotein receptor (*LDLR*) gene region harbors a DMR. Age-related methylation changes that were associated with lower expression levels of *LDLR* in the old sample (right panel, blue and red bars indicates expression in the young and old epidermis samples, respectively), which may promote the formation of Xanthelasma, a dermatological lesion often found in the elderly population.

To further characterize the DMRs, we used the available ENCODE data [27,28] for normal human epidermal keratinocytes. The results showed that DMRs that were hypermethylated in the old sample were enriched for H3K27me3 and H3K4me3, the defining chromatin marks of poised promoters. In contrast, hypomethylated DMRs that were hypomethylated in the old sample were enriched for H3K27Ac and also H3K4me1 (Figure 4A), which represent established marks of enhancers. We then used the available ChromHMM annotation for normal human epidermal keratinocytes [29] to assign our DMR datasets to defined chromatin states (Additional file 3: Table S3). Subsequent data analysis showed a distinct enrichment of DMRs in promoters and enhancers (Figure 4B), which confirms and expands previous observations on age-related methylation changes [13,14]. Furthermore, enhancer-associated DMRs showed a strong bias towards age-related hypomethylation (Figure 4B), which might reflect age-related activation of enhancers. Interestingly, active promoters showed no enrichment for DMRs (Figure 4B), which is again consistent with the comparably small effect size for age-related differential gene expression (Figure 1A).

A visual inspection of DMRs provided further insight into their characteristic features. For example, the ERBB receptor feedback inhibitor 1 (ERRFI1) promoter region harbors a DMR in the shore region of the promoter-associated CpG island (Figure 4C). ERRFI1 is required for proper epidermal homeostasis [30] and was found to be hypermethylated and expressed at lower levels in old epidermis samples (Figure 4C). Another example for a DMR was identified in the low-density lipoprotein receptor (*LDLR*) gene region (Figure 4D), and was located in an annotated active enhancer element (Additional file 4: Figure S1). The DMR showed complex, but coordinated age-related methylation changes that were associated with lower expression levels of *LDLR* in the old sample (Figure 4D). Defects in the *LDLR* gene are the cause of familial hypercholesterolemia, which underlies the formation of Xanthelasma [31], a dermatological lesion often found in the elderly population. These examples illustrate how age-related methylation changes can have relatively subtle, but significant expression changes on genes that are relevant for proper skin homeostasis. Further experiments will be required to determine the functional relevance of individual differentially methylated and expressed genes for the aging phenotype.

## Discussion

Whole-genome bisulfite sequencing represents a powerful method to generate genome-wide methylation maps at single-base resolution [24,32]. This approach was recently used to characterize the methylomes of purified CD4^+^ T-cells from a newborn and a centenarian [15]. The results showed a pronounced destabilization of the aging epigenome, which was characterized by a widespread loss of methylation marks. Our analysis failed to detect any quantitative differences in the global methylation levels of young and old individuals. This can most likely be attributed to the different tissues used in both studies: while our analysis is based on primary epidermis samples, Heyn et al. [15] used purified CD4^+^ T-cells. Based on the specific features of T-cell differentiation and priming, it is conceivable that the epigenetic program of these cells is characterized by a particularly high degree of plasticity.

On a global level, the transcriptomes and methylomes of the young and old epidermis appeared to be substantially similar. This is an important finding, because it illustrates the fundamental stability of the tissue-specific methylation landscape, which is required for the stable maintenance of cell type identity. Age-related methylation changes were limited to specific local alterations, which confirms and expands our previous observations [16]. With a size of 100–150 bp, age-related DMRs were considerably smaller than other known structures of the human methylome, such as DNA methylation valleys [3] and partially methylated domains [23-25,33,34], which extend over tens and hundreds of kilobases of DNA sequence, respectively. Further analysis will be required to understand the epigenetic regulatory function(s) of these elements.

Interestingly, our data also suggest that a significant fraction of the DMRs might represent enhancers that become aberrantly methylated during aging. The methylation status of enhancer-associated CpG dinucleotides has recently been described to be closely associated with epigenetic gene deregulation in human cancers [35,36]. Our data identify similar elements at single-base resolution and suggest that the differential methylation of enhancers might be involved in age-related gene deregulation.

Finally, our analysis identified several examples for hypermethylated DMRs in promoter regions. Promoter hypermethylation has been closely associated with gene silencing and plays an important role in the etiology of human tumors [37,38]. Furthermore, our data indicate an association of hypermethylated DMRs with bivalent chromatin structures. Similar results have been described in independent studies investigating age-related methylation changes in other tissues [13,14]. Bivalent chromatin modifications are a specific feature of stem cells and are not usually found in differentiated tissues like epidermis. The particular enrichment of age-related hypermethylation in promoters that are annotated as “bivalent” might therefore reflect epigenetic changes in aging epidermal stem cells, and may underpin the decreased regenerative capacity of aging stem cells [39].

## Conclusions

Age-related DNA methylation changes have been described in various studies but their defining features and functional significance remain to be established. Our results show that the age-related genome-wide loss of DNA methylation observed in T-cells [15] is not conserved in the human epidermis, suggesting that the core developmental program of at least some human tissues is maintained through the aging process. Furthermore, our study represents the first to use transcriptome sequencing to explore the consequences of age-related epigenetic changes. Our results identified limited transcriptional changes that may underpin the aging phenotype. Finally, we identified several hundred highly localized elements with robust methylation differences between young and old. Interestingly, these elements were enriched for gene regulatory chromatin marks and many of them were associated with promoters and enhancers. Our study thus provides an important mechanistic framework for understanding age-related epigenetic deregulation.

## Methods

### Sample preparation for sequencing

Epidermal suction blister samples were collected according to the current version of the Declaration of Helsinki and the guideline of the International Conference on Harmonization Good Clinical Practice (ICH GCP) was observed as applicable to a non-drug study. All volunteers provided written, informed consent. Suction blister samples were obtained at the study center of Beiersdorf AG and approved by the Beiersdorf AG Legal Review Board. Briefly, epidermis samples were detached from the forearms of 10 healthy female volunteers by applying a negative pressure of 150–250 mmHg for 2 h. Subsequently, suction blister roofs were taken and immediately stored in liquid nitrogen. For nucleic acid isolation, suction blister samples were washed in DPBS (Cambrex, Verviers, Belgium) and homogenized using a TissueLyser (Retsch, Haan, Germany). DNA and RNA from suction blister samples were isolated using the QIAamp DNA Investigator Kit (Qiagen, Hilden, Germany) and RNeasy Fibrous Tissue Kit (Qiagen), respectively, according to the manufacturer’s instructions. The Poly(A)Purist MAG Kit (Ambion, Darmstadt, Germany) was used for mRNA selection.

### Sequencing

Library preparation for bisulfite sequencing was performed as described previously [40]. Transcriptome sequencing libraries were prepared using the TruSeq RNA Sample Preparation Kit (Illumina, San Diego, USA), according to the manufacturer’s instructions. Paired-end sequencing was performed on an Illumina HiSeq system with read lengths of 105 base pairs and an average insert size of 200 bp.

### Transcriptome mapping and quantification of differential expression

RNA-seq reads were trimmed to a maximal length of 80 bp and stretches of bases having a quality score <30 at the ends of the reads were removed. Reads were mapped using Tophat 2.0.6 [19]. As reference sequence for the transcriptome mapping we used the current assembly of the human genome (hg19). Differential expression was quantified using DESeq 1.10.1 [20] applying the built-in procedures for library normalization and estimation of variance and with Cuffdiff 2.0 [21]. The resulting *P* values were subjected to multiple testing correction using built-in functions available in DESeq and Cuffdiff, respectively. Genes with a q value smaller than 0.05 were considered as differentially expressed.

### Pathway analysis

Pathway analysis of differentially expressed genes was performed using IPA (Ingenuity Systems), using a *P* value cutoff of <0.05 on differential expression and a log fold-change of at least 0.263, corresponding to a minimum expression change of 30% between young and old samples.

### qRT-PCR

Total RNA was isolated from suction blister samples (n = 18) using the RNeasy Fibrous Tissue Kit (Qiagen) according to the manufacturer’s instructions. After reverse transcription with the High Capacity cDNA Reverse Transcription Kit (Applied Biosystems, Darmstadt, Germany), samples were analyzed by TaqMan-PCR using the 7900HT Fast-Real-Time PCR System (Applied Biosystems). The following assays were used, according to the manufacturer’s recommendations: CTGF (Hs01026927_g1), SPRR1A (Hs00954595_s1), SPRR1B (Hs00234164_m1), KRT16 (Hs00373910_g1), KRT17 (Hs01588578_m1), VCL (Hs00247826_m1), HAS3 (Hs00193436_m1), FDZ10 (Hs00273077_s1), NPR3 (Hs00168558_m1), VPS37B (Hs00226582_m1), ERRFI1 (Hs00219060_m1), PLIN2 (Hs00605340_m1), RHPN2 (Hs00369111_m1), RGMA (Hs00297192_m1), PI4KB (Hs01090927_m1). Data were analyzed utilizing the Sequence detector version 2.3 software supplied with the 7900 Sequence Detector and RQ Manager 1.2. Quantification was achieved using the ΔCt method which indicates expression of the target gene relative to an endogenous reference (GAPDH; Hs99999905_m1). ΔCt values were averaged for all replicates of a gene/subject combination and for every gene the mean value over all subjects was subtracted, thus adjusting the average gene-specific ΔCt value to zero. The processed ΔCt values were visualized as heat maps. ΔCt values exceeding a threshold of 2.5 were set to 2.5.

### Bisulfite mapping and methylation calling

Reads were trimmed to a maximal length of 80 bp and stretches of bases having a quality score <30 at the ends of the reads were removed. Reads were mapped using BSMAP 2.02 [41]. As a reference sequence for the bisulfite mapping we used the current assembly of the human genome (hg19). Only reads mapping with both partners of the read pairs at the correct distance were used. The CpG-specificity was calculated by determining the number of cytosines called in all mapped reads at all non-CpG positions and dividing it by the number of all bases in all mapped reads at all non-CpG positions. Methylation ratios were determined using a Python script (methratio.py) distributed together with the BSMAP package. For both the forward and reverse strands, all cytosine bases in CG context were called independently.

### Identification and characterization of DMRs

Fisher’s exact test was used to identify 3,004,806 CpG dinucleotides with a statistically significant (*P* <0.05) difference in their methylation ratios between the young and the old datasets. Differentially methylated CpGs were subsequently collapsed to identify regions of local, coordinated methylation change. DMRs were defined as clusters of ≥8 DMCs with a distance of ≤50 bp between neighboring DMCs and a net region-wide methylation change of ≥8 DMCs. Only DMRs with an average sequencing coverage of ≥8 and methylation difference of ≥10% were used for further analysis. ChIP-seq peaks of histone modification marks in NHEK cells were downloaded from the ENCODE website [29] and averaged in 50 bp windows surrounding the DMR sites. Loess smoothing with a span of 10% was applied. For the enrichment analysis of DMRs in defined chromatin states, the observed frequency (%) of DMRs centered in given states (ChromHMM annotation data in NHEK cells, ENCODE website) was divided by the genomic frequency of the state.

### Data access

Sequencing data have been deposited in the GEO database under the accession number GSE46487.

## Abbreviations

CTGF: Connective tissue growth factor; DMC: Differentially methylated CpG; DMR: Differentially methylated regions; ERRFI1: ERBB receptor feedback inhibitor 1; LDLR: Low-density lipoprotein receptor.

## Competing interests

MW, SH and JS are employees of Beiersdorf AG. FL received consultation fees from Beiersdorf AG.

## Authors’ contributions

GR, SH, DA, PPK, LK and AH analyzed the data. JS performed experiments. SH and MW contributed the epidermis samples. MW and FL conceived the study. FL wrote the paper. All authors read and approved the final manuscript.

## Supplementary Material

Additional file 1: Table S1 Samples used for sequencing. The table shows a complete overview of all epidermis samples used for methylome and transcriptome sequencing. Click here for file

Additional file 2: Table S2 List of differentially expressed genes. Differentially expressed genes were identified by DESeq using built-in procedures for library normalization and estimation of variance. baseMean is the average of baseMean (young) and baseMean (old) and indicates the number of fragments per gene after library normalization, *P* values were subjected to multiple testing correction using built-in functions available in DESeq, leading to adjusted *P* values (“padj”). Genes with an adjusted *P* value <0.05 were considered as differentially expressed and are included in this table. Click here for file

Additional file 3: Table S3 Association of DMRs with ChromHMM segments. The table shows the results obtained from ChromHMM segmentation of the human genome sequence, using ENCODE data for normal human keratinocytes. Click here for file

Additional file 4: Figure S1 Epigenomic analysis of the *LDLR* gene region. UCSC Genome Browser tracks for DNA methylation and various histone marks, based on ENCODE data for normal human keratinocytes. Click here for file

## References

[B1] Klose RJ Bird AP Genomic DNA methylation: the mark and its mediators Trends Biochem Sci 2006 31 2 89 97 10.1016/j.tibs.2005.12.008 16403636

[B2] Kouzarides T Chromatin modifications and their function Cell 2007 128 4 693 705 10.1016/j.cell.2007.02.005 17320507

[B3] Xie W Schultz MD Lister R Hou Z Rajagopal N Ray P Whitaker JW Tian S Hawkins RD Leung D Yang H Wang T Lee AY Swanson SA Zhang J Zhu Y Kim A Nery JR Urich MA Kuan S Yen CA Klugman S Yu P Suknuntha K Propson NE Chen H Edsall LE Wagner U Li Y Ye Z Epigenomic analysis of multilineage differentiation of human embryonic stem cells Cell 2013 153 5 1134 1148 10.1016/j.cell.2013.04.022 23664764 PMC3786220

[B4] Gifford CA Ziller MJ Gu H Trapnell C Donaghey J Tsankov A Shalek AK Kelley DR Shishkin AA Issner R Zhang X Coyne M Fostel JL Holmes L Meldrim J Guttman M Epstein C Park H Kohlbacher O Rinn J Gnirke A Lander ES Bernstein BE Meissner A Transcriptional and epigenetic dynamics during specification of human embryonic stem cells Cell 2013 153 5 1149 1163 10.1016/j.cell.2013.04.037 23664763 PMC3709577

[B5] Mohn F Schubeler D Genetics and epigenetics: stability and plasticity during cellular differentiation Trends Genet 2009 25 3 129 136 10.1016/j.tig.2008.12.005 19185382

[B6] Bergman Y Cedar H DNA methylation dynamics in health and disease Nat Struct Mol Biol 2013 20 3 274 281 10.1038/nsmb.2518 23463312

[B7] Li E Bestor TH Jaenisch R Targeted mutation of the DNA methyltransferase gene results in embryonic lethality Cell 1992 69 915 926 10.1016/0092-8674(92)90611-F 1606615

[B8] Okano M Bell DW Haber DA Li E DNA methyltransferases Dnmt3a and Dnmt3b are essential for *de novo* methylation and mammalian development Cell 1999 99 247 257 10.1016/S0092-8674(00)81656-6 10555141

[B9] Feinberg AP Phenotypic plasticity and the epigenetics of human disease Nature 2007 447 7143 433 440 10.1038/nature05919 17522677

[B10] Fraga MF Ballestar E Paz MF Ropero S Setien F Ballestar ML Heine-Suner D Cigudosa JC Urioste M Benitez J Boix-Chornet M Sanchez-Aguilera A Ling C Carlsson E Poulsen P Vaag A Stephan Z Spector TD Wu YZ Plass C Esteller M Epigenetic differences arise during the lifetime of monozygotic twins Proc Natl Acad Sci USA 2005 102 30 10604 10609 10.1073/pnas.0500398102 16009939 PMC1174919

[B11] Winnefeld M Lyko F The aging epigenome: DNA methylation from the cradle to the grave Genome Biol 2012 13 7 165 22839706 10.1186/gb-2012-13-7-165 PMC3491376

[B12] Christensen BC Houseman EA Marsit CJ Zheng S Wrensch MR Wiemels JL Nelson HH Karagas MR Padbury JF Bueno R Sugarbaker DJ Yeh RF Wiencke JK Kelsey KT Aging and environmental exposures alter tissue-specific DNA methylation dependent upon CpG island context PLoS Genet 2009 5 8 e1000602 10.1371/journal.pgen.1000602 19680444 PMC2718614

[B13] Rakyan VK Down TA Maslau S Andrew T Yang TP Beyan H Whittaker P McCann OT Finer S Valdes AM Leslie RD Deloukas P Spector TD Human aging-associated DNA hypermethylation occurs preferentially at bivalent chromatin domains Genome Res 2010 20 4 434 439 10.1101/gr.103101.109 20219945 PMC2847746

[B14] Teschendorff AE Menon U Gentry-Maharaj A Ramus SJ Weisenberger DJ Shen H Campan M Noushmehr H Bell CG Maxwell AP Savage DA Mueller-Holzner E Marth C Kocjan G Gayther SA Jones A Beck S Wagner W Laird PW Jacobs IJ Widschwendter M Age-dependent DNA methylation of genes that are suppressed in stem cells is a hallmark of cancer Genome Res 2010 20 4 440 446 10.1101/gr.103606.109 20219944 PMC2847747

[B15] Heyn H Li N Ferreira HJ Moran S Pisano DG Gomez A Diez J Sanchez-Mut JV Setien F Carmona FJ Puca AA Sayols S Pujana MA Serra-Musach J Iglesias-Platas I Formiga F Fernandez AF Fraga MF Heath SC Valencia A Gut IG Wang J Esteller M Distinct DNA methylomes of newborns and centenarians Proc Natl Acad Sci USA 2012 109 26 10522 10527 10.1073/pnas.1120658109 22689993 PMC3387108

[B16] Gronniger E Weber B Heil O Peters N Stab F Wenck H Korn B Winnefeld M Lyko F Aging and chronic sun exposure cause distinct epigenetic changes in human skin PLoS Genet 2010 6 5 e1000971 10.1371/journal.pgen.1000971 20523906 PMC2877750

[B17] Zouboulis CC Makrantonaki E Clinical aspects and molecular diagnostics of skin aging Clin Dermatol 2010 29 1 3 14 21146726 10.1016/j.clindermatol.2010.07.001

[B18] Sen GL Reuter JA Webster DE Zhu L Khavari PA DNMT1 maintains progenitor function in self-renewing somatic tissue Nature 2010 463 7280 563 567 10.1038/nature08683 20081831 PMC3050546

[B19] Trapnell C Pachter L Salzberg SL TopHat: discovering splice junctions with RNA-Seq Bioinformatics 2009 25 9 1105 1111 10.1093/bioinformatics/btp120 19289445 PMC2672628

[B20] Anders S Huber W Differential expression analysis for sequence count data Genome Biol 2012 11 10 R106 20979621 10.1186/gb-2010-11-10-r106 PMC3218662

[B21] Trapnell C Hendrickson DG Sauvageau M Goff L Rinn JL Pachter L Differential analysis of gene regulation at transcript resolution with RNA-seq Nat Biotechnol 2012 31 1 46 53 10.1038/nbt.2450 23222703 PMC3869392

[B22] Quan T Shao Y He T Voorhees JJ Fisher GJ Reduced expression of connective tissue growth factor (CTGF/CCN2) mediates collagen loss in chronologically aged human skin J Invest Dermatol 2010 130 2 415 424 10.1038/jid.2009.224 19641518 PMC2877594

[B23] Raddatz G Gao Q Bender S Jaenisch R Lyko F Dnmt3a protects active chromosome domains against cancer-associated hypomethylation PLoS Genet 2012 8 12 e1003146 10.1371/journal.pgen.1003146 23284304 PMC3527206

[B24] Lister R Pelizzola M Dowen RH Hawkins RD Hon G Tonti-Filippini J Nery JR Lee L Ye Z Ngo QM Edsall L Antosiewicz-Bourget J Stewart R Ruotti V Millar AH Thomson JA Ren B Ecker JR Human DNA methylomes at base resolution show widespread epigenomic differences Nature 2009 462 7271 315 322 10.1038/nature08514 19829295 PMC2857523

[B25] Hon GC Hawkins RD Caballero OL Lo C Lister R Pelizzola M Valsesia A Ye Z Kuan S Edsall LE Camargo AA Stevenson BJ Ecker JR Bafna V Strausberg RL Simpson AJ Ren B Global DNA hypomethylation coupled to repressive chromatin domain formation and gene silencing in breast cancer Genome Res 2012 22 2 246 258 10.1101/gr.125872.111 22156296 PMC3266032

[B26] Ziller MJ Gu H Muller F Donaghey J Tsai LT Kohlbacher O De-Jager PL Rosen ED Bennett DA Bernstein BE Gnirke A Meissner A Charting a dynamic DNA methylation landscape of the human genome Nature 2013 500 7463 477 481 10.1038/nature12433 23925113 PMC3821869

[B27] Ernst J Kellis M Discovery and characterization of chromatin states for systematic annotation of the human genome Nat Biotechnol 2010 28 8 817 825 10.1038/nbt.1662 20657582 PMC2919626

[B28] Ernst J Kellis M ChromHMM: automating chromatin-state discovery and characterization Nat Methods 2012 9 3 215 216 10.1038/nmeth.1906 22373907 PMC3577932

[B29] Consortium EP A user’s guide to the encyclopedia of DNA elements (ENCODE) PLoS Biol 2011 9 4 e1001046 10.1371/journal.pbio.1001046 21526222 PMC3079585

[B30] Ferby I Reschke M Kudlacek O Knyazev P Pante G Amann K Sommergruber W Kraut N Ullrich A Fassler R Klein R Mig6 is a negative regulator of EGF receptor-mediated skin morphogenesis and tumor formation Nat Med 2006 12 5 568 573 10.1038/nm1401 16648858

[B31] Ishibashi S Goldstein JL Brown MS Herz J Burns DK Massive xanthomatosis and atherosclerosis in cholesterol-fed low density lipoprotein receptor-negative mice J Clin Invest 1994 93 5 1885 1893 10.1172/JCI117179 8182121 PMC294295

[B32] Lister R O’Malley RC Tonti-Filippini J Gregory BD Berry CC Millar AH Ecker JR Highly integrated single-base resolution maps of the epigenome in Arabidopsis Cell 2008 133 3 523 536 10.1016/j.cell.2008.03.029 18423832 PMC2723732

[B33] Lister R Pelizzola M Kida YS Hawkins RD Nery JR Hon G Antosiewicz-Bourget J O’Malley R Castanon R Klugman S Downes M Yu R Stewart R Ren B Thomson JA Evans RM Ecker JR Hotspots of aberrant epigenomic reprogramming in human induced pluripotent stem cells Nature 2011 471 7336 68 73 10.1038/nature09798 21289626 PMC3100360

[B34] Berman BP Weisenberger DJ Aman JF Hinoue T Ramjan Z Liu Y Noushmehr H Lange CP Van-Dijk CM Tollenaar RA Van Den-Berg D Laird PW Regions of focal DNA hypermethylation and long-range hypomethylation in colorectal cancer coincide with nuclear lamina-associated domains Nat Genet 2012 44 1 40 46 22120008 10.1038/ng.969 PMC4309644

[B35] Aran D Sabato S Hellman A DNA methylation of distal regulatory sites characterizes dysregulation of cancer genes Genome Biol 2013 14 3 R21 10.1186/gb-2013-14-3-r21 23497655 PMC4053839

[B36] Aran D Hellman A DNA methylation of transcriptional enhancers and cancer predisposition Cell 2013 154 1 11 13 10.1016/j.cell.2013.06.018 23827668

[B37] Esteller M Epigenetics in cancer N Engl J Med 2008 358 11 1148 1159 10.1056/NEJMra072067 18337604

[B38] Baylin SB Jones PA A decade of exploring the cancer epigenome – biological and translational implications Nat Rev Cancer 2011 11 10 726 734 10.1038/nrc3130 21941284 PMC3307543

[B39] Sharpless NE DePinho RA How stem cells age and why this makes us grow old Nat Rev Mol Cell Biol 2007 8 9 703 713 10.1038/nrm2241 17717515

[B40] Lyko F Foret S Kucharski R Wolf S Falckenhayn C Maleszka R The honey bee epigenomes: differential methylation of brain DNA in queens and workers PLoS Biol 2010 8 11 e1000506 10.1371/journal.pbio.1000506 21072239 PMC2970541

[B41] Xi Y Li W BSMAP: whole genome bisulfite sequence MAPping program BMC Bioinformatics 2009 10 232 10.1186/1471-2105-10-232 19635165 PMC2724425

