# Supplementary material for: Aging is associated with highly defined epigenetic changes in the human epidermis
Source: Epigenetics Chromatin. 2013 Oct 31;6:36. doi: 10.1186/1756-8935-6-36 (PMC3819645; doi:10.1186/1756-8935-6-36)
Supplement: Additional file 1: Table S1 — Samples used for sequencing. The table shows a complete overview of all epidermis samples used for methylome and transcriptome sequencing. [file 1756-8935-6-36-S1.doc]

**Additional file 1. Samples used for sequencing.**

| **pool** | **sample no.** | **tissue** | **gender** | **age (y.)** |
| --- | --- | --- | --- | --- |
| old | 1 | epidermis | female | 71 |
|  | 4 | epidermis | female | 70 |
|  | 9 | epidermis | female | 72 |
|  | 10 | epidermis | female | 73 |
|  | 14 | epidermis | female | 75 |
|  |  |  |  |  |
| young | 8 | epidermis | female | 24 |
|  | 11 | epidermis | female | 21 |
|  | 12 | epidermis | female | 20 |
|  | 15 | epidermis | female | 18 |
|  | 16 | epidermis | female | 21 |
