# Supplementary material for: Aging is associated with highly defined epigenetic changes in the human epidermis
Source: Epigenetics Chromatin. 2013 Oct 31;6:36. doi: 10.1186/1756-8935-6-36 (PMC3819645; doi:10.1186/1756-8935-6-36)
Supplement: Additional file 3: Table S3 — Association of DMRs with ChromHMM segments. The table shows the results obtained from ChromHMM segmentation of the human genome sequence, using ENCODE data for normal human keratinocytes. [file 1756-8935-6-36-S3.doc]

**Additional file 3. Association of DMRs with ChromHMM segmets.**

| **segment** | **% of genome** | **no. of hyperDMRs** | **no of hypoDMRs** |
| --- | --- | --- | --- |
| 1_Active_Promoter | 4.05 | 78 | 31 |
| 2_Weak_Promoter | 1.52 | 87 | 51 |
| 3_Poised_Promoter | 1.47 | 121 | 43 |
| 4_Strong_Enhancer | 1.33 | 44 | 61 |
| 5_Strong_Enhancer | 1.31 | 24 | 45 |
| 6_Weak_Enhancer | 1.38 | 46 | 41 |
| 7_Weak_Enhancer | 1.90 | 29 | 38 |
| 8_Insulator | 0.92 | 31 | 23 |
| 9_Txn_Transition | 0.98 | 22 | 26 |
| 10_Txn_Elongation | 6.27 | 65 | 37 |
| 11_Weak_Txn | 14.94 | 125 | 83 |
| 12_Repressed | 7.64 | 327 | 147 |
| 13_Heterochrom/lo | 55.65 | 393 | 305 |
| 14_Repetitive/CNV | 0.19 | 14 | 22 |
| 15_Repetitive/CNV | 0.14 | 139 | 63.0 |

The human genome sequence was segmented by ChromHMM, using ENCODE data for normal human epidermal keratinocytes.
