# Supplementary figures and images for: Aging is associated with highly defined epigenetic changes in the human epidermis
Source: Epigenetics Chromatin. 2013 Oct 31;6:36. doi: 10.1186/1756-8935-6-36 (PMC3819645; doi:10.1186/1756-8935-6-36)

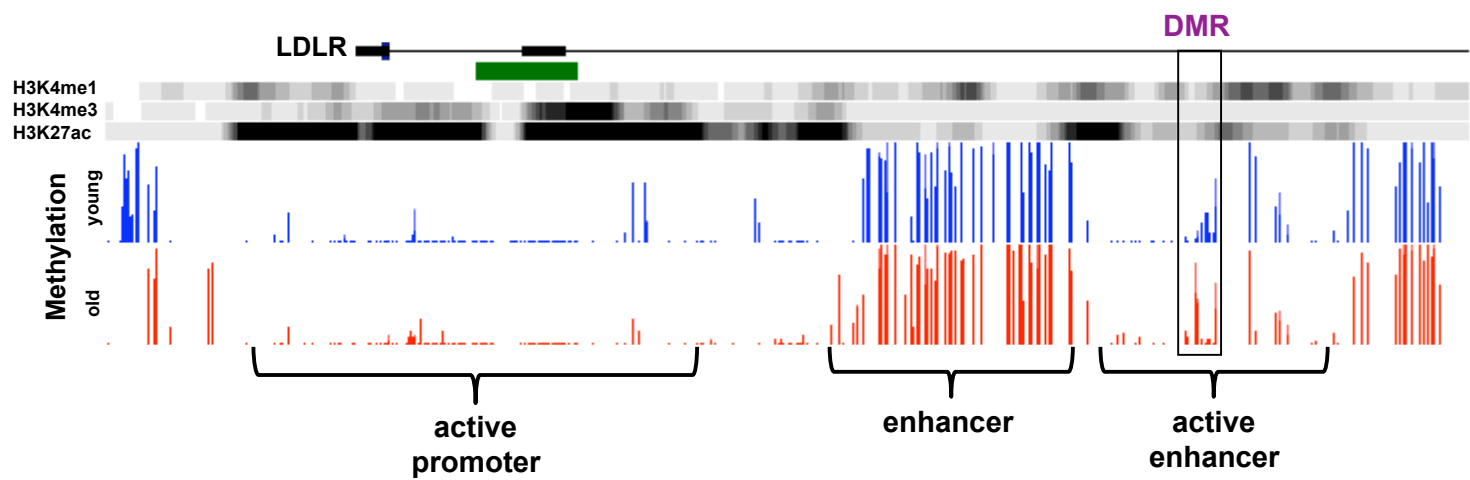

Supplement: Additional file 4: Figure S1 — Epigenomic analysis of the LDLR gene region. UCSC Genome Browser tracks for DNA methylation and various histone marks, based on ENCODE data for normal human keratinocytes. [file 1756-8935-6-36-S4.pdf]
